# Abnormal coagulation parameters are associated with poor prognosis in patients with novel coronavirus pneumonia

**DOI:** 10.1111/jth.14820

**Published:** 2020-04-29

**Authors:** Deepa R. J. Arachchillage, Mike Laffan

**Affiliations:** ^1^ Centre for Haematology Imperial College London London UK; ^2^ Department of Haematology Imperial College Healthcare NHS Trust London UK; ^3^ Haematology Royal Brompton Hospital London UK

A recently published article by Dr. Tang and colleagues on “Abnormal coagulation parameters are associated with poor prognosis in patients with novel coronavirus pneumonia” highlighted that disseminated intravascular coagulation is common severe respiratory failure patients with Novel Coronavirus (Covid‐19).^1^ Covid‐19 infection leading to pneumonia and severe acute respiratory distress syndrome (ARDS) was first reported in Wuhan, Hubei Province, China, and has subsequently spread to almost all other countries in the world. On November 3, 2020, the World Health Organization declared the Covid‐19 outbreak a global pandemic. Patients with severe illness may develop dyspnea and hypoxemia within 1 week after onset of the disease, which may quickly progress to ARDS or end‐organ failure.

Tang and colleagues reported in their retrospective study of 183 consecutive patients with confirmed Covid‐19 pneumonia at Tongji hospital in China,^1^ showing that patients who died (11.5%) had significantly higher D‐dimer and fibrin degradation product levels, longer prothrombin time (PT) and activated partial thromboplastin time at presentation compared to those who survived. Of nonsurvivors, 71.4% met the International Society on Thrombosis and Haemostasis diagnostic criteria for overt disseminated intravascular coagulation (DIC) (≥5 points)^2^ (Table 1) compared with 0.6% of survivors. The median time from admission to DIC was 4 days (range, 1‐12 days). It was evident that abnormal coagulation parameters (prolonged PT and raised D‐dimer) are predictors of a poor prognosis and may be important therapeutic targets.

**Table 1:** International Society on Thrombosis and Haemostasis diagnostic criteria for disseminated intravascular coagulation

Parameter	Score
Platelet count	
>100 × 10^9^/L	0
50‐100 × 10^9^/L	1
<50 × 10^9^/L	2
D dimer	
No increase	0
Moderate increase (1‐10 times upper limit of normal)	2
Strong increase (>10 times upper limit of normal)	3
Fibrinogen	
>1.0 g/L	0
≤1.0 g/L	1
Prothrombin time prolongation	
<3 s	0
3‐6 s	1
>6 s	2
Overt disseminated intravascular coagulation	≥ 5

Note

Adapted from Taylor et al.^3^

In another study with 201 patients, 84 patients developed ARDS. Patients who developed ARDS had significantly higher PT (median [interquartile range]) of 11.7 seconds (11.10 to −12.4 vs 10.6 seconds [10.1‐11.5], *P* < .001, and D‐dimer of 1.16 µg/mL [0.46‐5.37] vs 0.52 µg/mL [0.33‐0.93]), *P* < .001 at presentation compared with those did not develop ARDS.^1^ Of 84 patients who developed ARDS, 52.8% (44/84) patients died and these patients had significantly higher D‐dimer levels (3.95 µg/mL [1.15 to −10.96]) compared with those who survived (0.49 µg/mL [0.31 to −1.18], *P* = .001.^3^ Interestingly, thrombocytopenia does not seem to be common and was present in only 37/201(18.8%) compared with >50% patients presenting with ARDS from other causes such as bacterial and other viral infections.^4^

We would like to highlight the implications of Coagulation abnormalities associated with Covid‐19 in patients receiving venovenous extracorporeal membrane oxygenation (VV‐ECMO) as the high proportion of Covid‐19 patients developing ARDS means that many will also require VV‐ECMO. We have previously reported on the high frequency of intracranial hemorrhage in patients receiving VV‐ECMO and so the high frequency in COVID19 is of concern.^5^ However, the lack of thrombocytopenia may be beneficial as we found this to be a risk factor for ICH.^5^ The studies cited here have led to suggestions that DIC may be a useful prognostic marker, but the anticoagulation required for VV‐ECMO and the activation of coagulation from artificial surfaces may confound interpretation. In particular, a sudden rise in D‐dimer may be because of pump head thrombosis.

In summary, patients with severe Covid‐19 are at greater risk of DIC, which may be further complicated by the effects of the ECMO circuit and the combination may increase thrombo‐hemorrhagic morbidity. We expect that careful interpretation of coagulation abnormalities and systemic anticoagulation will be required, and standard protocols may need adapting to this new disorder.

## CONFLICT OF INTEREST

The authors state that they have no conflict of interest.

## AUTHOR CONTRIBUTIONS

DRJ Arachchillage wrote the first draft. M Laffan reviewed the manuscript, and both authors approved the final manuscript.

## References

[1] Tang N , Li D , Wang X , Sun Z . Abnormal coagulation parameters are associated with poor prognosis in patients with novel coronavirus pneumonia. J Thromb Haemost. 2020;18(4):844‐847. 32073213 10.1111/jth.14768 PMC7166509

[2] Taylor FB Jr , Toh CH , Hoots WK , Wada H , Levi M . Towards definition, clinical and laboratory criteria, and a scoring system for disseminated intravascular coagulation. Thromb Haemost. 2001;86:1327‐1330. 11816725

[3] Wu C , Chen X , Cai Y , et al. Risk factors associated with acute respiratory distress syndrome and death in patients with coronavirus disease 2019 pneumonia in Wuhan, China. JAMA Intern Med. 2020. 10.1001/jamainternmed.2020.0994. [Epub ahead of print] PMC7070509 32167524

[4] Arachchillage DRJ , Laffan M , Khanna S , et al. Frequency of thrombocytopenia and heparin‐induced thrombocytopenia in patients receiving extracorporeal membrane oxygenation compared with cardiopulmonary bypass and the limited sensitivity of pretest probability score. Crit Care Med. 2020;48:e371‐e379. 32058356 10.1097/CCM.0000000000004261

[5] Arachchillage DRJ , Passariello M , Laffan M , et al. Intracranial hemorrhage and early mortality in patients receiving extracorporeal membrane oxygenation for severe respiratory failure. Semin Thromb Hemost. 2018;44(3):276‐286. 29566407 10.1055/s-0038-1636840

